# Differentiating Endurance-and Speed-Adapted Types of Elite and World Class Milers According to Biomechanical, Pacing and Perceptual Responses during a Sprint Interval Session

**DOI:** 10.3390/ijerph18052448

**Published:** 2021-03-02

**Authors:** Arturo Casado, Andrew Renfree, José Carlos Jaenes-Sánchez, Víctor Cuadrado-Peñafiel, Pedro Jiménez-Reyes

**Affiliations:** 1 Centre for Sport Studies, Rey Juan Carlos University, Camino del Molino s/n, 28943 Madrid, Spain; pedro.jimenezr@urjc.es; 2 Insititute of Sport and Exercise Science, University of Worcester, Worcester WR2 6AJ, UK; a.renfree@worc.ac.uk; 3 Department of Social Anthropology & Basic Psychology & Health, Pablo de Olavide University, 41704 Sevilla, Spain; jcjaesan@upo.es; 4 Education Faculty, Autónoma University of Madrid, 28049 Madrid, Spain; victor.cuadrado@uam.es

**Keywords:** athletics, exercise performance, perceptions, coaching

## Abstract

The aim was to compare pacing, biomechanical and perceptual responses between elite speed-and endurance-adapted milers during a sprint interval training session (SIT). Twenty elite and world-class middle-distance runners (male: *n* = 16, female: n = 4; 24.95 ± 5.18 years; 60.89 ± 7 kg) were classified as either speed- or endurance-adapted milers according to their recent performances at 800 m or longer races than 1500 m (10 subjects per group). Participants performed 10 repetitions of 100 m sprints with 2 min of active recovery between each, and performance, perceptual and biomechanical responses were collected. The difference between accumulated times of the last and the first five repetitions was higher in speed-adapted milers (ES = 1.07) displaying a more positive pacing strategy. A higher coefficient of variation (CV%) was displayed across the session by speed-adapted milers in average repetition time, contact time, and affective valence (ES ≥ 1.15). Speed-adapted milers experienced lower rates of valence after the 4th repetition excepting at the 8th repetition (ES ≥ 0.99). Speed-adapted milers may need to display a more positive pacing profile than endurance-adapted milers and, therefore, would experience lower levels of affective valence and a more rapid increase of ground contact time during a SIT.

## 1. Introduction

The 800 m, 1500 m, and 3000 m events are considered middle-distance running races and at the elite level are typically completed in between 1.6 and 10 min [1], meaning a range of physiological and biomechanical qualities, which determines performance in these events. Although middle-distance running events are characterized by a high relative contribution from the aerobic energy system [2] and performance in these events is highly correlated with the speed at which maximal oxygen uptake is achieved (vVO_2max_) [3], the high speeds at which elite races are completed demand high levels of biomechanical power output and a well-developed anaerobic capacity [4]. However, 1500 m and mile runners (milers) can approach the event from either the 800 m or 3000 m ends of the speed-endurance spectrum due to differing physiological adaptations achieved through specific-distance training or innate characteristics such as the individual’s muscle fiber [5]. Consequently, they may display different performance-related characteristics (i.e., a 800–1500 m runner would be able to produce greater power biomechanical output [6] whereas a 1500–3000 m runner would display a higher relative contribution of the aerobic energy system [2]. We can, therefore, classify these runners as either endurance-adapted or speed-adapted milers. In a similar vein, Nummela and Rusko [7] found a significantly greater aerobic contribution for endurance trained subjects compared with sprint trained participants during the first 30 s of 49 s of exhaustive treadmill running. Conversely, it has been suggested that 800 m runners are required to display high levels of power output early in the race, which are not required for success during the 3000 m event, whereas during the latest stages of the race, the relative contribution of the aerobic energy system increases [6].

Due to differing demands of the events, pacing strategies observed during 800 m world record races are mostly positive (the second half of the race is covered slower than the first) whereas in longer races such as 5000 m, runners display a fast end spurt [8,9]. From a biomechanical perspective, in middle-distance running races, high ground reaction forces are generated but they are lower than in sprint races [10]. Furthermore, a longer stride and a shorter contact time has been observed in middle-distance runners when compared to long-distance runners [11]. Whilst these biomechanical variables have not been found to discriminate across performance level in runners [12], it has been observed that during a repeated sprint ability (RSA) session, contact time and step length increased with fatigue and lower speed whereas step frequency decreased [13]. Accordingly, speed-adapted milers may be expected to display a more positive pacing profile when conducting a short interval training (SIT) session than endurance-adapted milers and, although changes in the biomechanical responses are to be expected throughout the session, these might also differ between the two groups. Furthermore, any difference in pacing profile displayed by the two types of milers may also result in differences in the progression of change of perceptual responses during the SIT session. For example, a greater change in rating of perceived exertion (RPE) across the session in the speed than in endurance-adapted runners might be expected. However, a recently proposed three-dimensional framework of centrally regulated and goal directed exercise behavior emphasizes the dynamic and complex interplay of sensory, affective, and cognitive processes that underpin perceived fatigability [14]. This framework more comprehensively accounted for perception–thinking–action coupling in response to psychophysiological distress than the traditional Gestalt concept of perceived exertion [14]. Therefore, another psychological variable that has been demonstrated to be implicated in the awareness of achievement of performance is core affect. Specifically, a more negative affective state or valence is associated with low performance [14,15]. Accordingly, speed-adapted milers may also display a greater change in affective valence than endurance-adapted runners across a SIT session due to their presumably more positive pacing strategy. The analysis of these variables in elite middle-distance runners may help coaches to make correct training decisions regarding the optimal approach that should be used for each type of runner. Therefore, the aim of this study was to compare the different performance, biomechanical and perceptual responses among elite speed- and endurance-adapted milers during a SIT session.

## 2. Materials and Methods

### 2.1. Participants

Twenty elite middle-distance runners (male: n = 16, female: n = 4; age = 24.95 ± 5.18 years old; body mass = 60.89 ± 7 kg; height = 174.7 ± 6.48 cm) were recruited from a professional middle-distance running group. All participants are currently active at national or international level by the time of writing the present article and 15 of them have been selected by their national federation to compete at international events. Two of the participants competed at two final races of the 2019 World Championships of Athletics. Runners competed at the 1500 m event regularly. Mean of their 1500 m best performance during the year preceding the study was 230.56 ± 10.88 s for males, and 266.76 ± 6.3 s for females. Participants completed 7.2 ± 1.4 training sessions per week. They had been systematically training for 7.7 ± 3.2 years. Neither physical limitations nor musculoskeletal injuries that could affect testing for at least six months prior to the test were reported. All participants provided written informed consent prior to participation in the experimental procedures. The study protocol adhered to the tenets of the Declaration of Helsinki and was approved by the Institutional Review Board of Pablo de Olavide University (935/CEIH/2019).

### 2.2. Experimental Design

An independent measures experimental design was employed involving assessment of performance, biomechanical and psychological variables during one session of high-intensity repetition running. Participants were divided in two groups of ten athletes according to their coach’s perceptions. The coach based this decision on the target event for the season, and the type of training being conducting (i.e., lower volume and higher intensity in the training of the milers who also were training for 800 m and higher volume and lower intensity in the training of the milers who also were training for either the 3000 m, 3000 m steeplechase, or 5000 m). Furthermore, this decision was further checked through an analysis of the difference in recent competitive performances in both shorter (i.e., 800 m) and longer (i.e., 3000 m, 3000 m steeplechase, and 5000 m) events than 1500 m. Fastest performance times achieved by participants during competition in the 12 months prior to testing were collected from the World Athletics open access website (www.worldathletics.org (accessed on 5 February 2021)) and transformed into International Association of Athletics Federations (IAAF) scores [16]. Participants were allocated to groups (speed- or endurance-adapted) depending on whether they achieved a “better” recent performance in either the shorter or longer events than 1500 m event.

Participants were requested to arrive for testing in a rested state, thereby having avoided intense exercise during the previous 48 h. They also were instructed to be in a fully hydrated state and having fasted for at least 3 h. These conditions were confirmed by athletes prior to the test. They were asked to prepare their training and diet for 48 h prior to the session, thereby simulating their typical routine before a high-intensity running session or competition. This session was completed at 11 a. m. on a synthetic indoor athletics running track. Temperature and humidity were constantly between 20 °C and 22 °C and between 35 and 40%. A standardized warm-up protocol was used by all participants, consisting of 15 min of running at a self-selected easy pace, 5 min of joint mobilization exercises, and two 30 m running accelerations. Subsequently, athletes performed 10 bouts of 100 m sprints at the highest possible speed with an active recovery period of 2 min between attempts in which they walked back to the starting point. Performance, biomechanical and perceptual responses were collected from participants across the session. Coefficient of variation for every measure collected from athletes during each repetition (CV%) was calculated using the mean and standard deviation (SD) in order to assess the variability in each variable across the SIT session. Specific distance length, number of repetitions, and recovery times between repetitions were set in order to induce a demand for high biomechanical outputs without a very high anaerobic glycolytic energy contribution, which also may allow for a sufficient contribution of the aerobic system by means of a high muscle O_2_ demand and greater reliance on oxidative metabolism [17,18].

### 2.3. Measures

#### 2.3.1. 100 m Sprint Time and Maximal Speed

Sprint times were recorded for both 100 m and 30–40 m distances using photocell timing gates (Polifemo Radio Light Racetime, Microgate, Bolzano, Italy). This intermediate distance was chosen because it has been reported that top speed during a maximal sprint is reached at this point [19]. Participants used a standing start, placing the leadoff foot 1 m behind the first timing gate. A standard crouched start position was adopted by participants. They placed the toes of their preferred leg just behind the start line. Once in position participants were asked to start the sprint when they would be ready for it. Athletes were instructed to accelerate maximally, thereby attempting to complete the sprint distance as fast as possible. Athletes wore spike shoes during the SIT.

#### 2.3.2. Biomechanical Variables

Ten meters of optoelectronic system (Optojump Next Microgate, Bolzano, Italy) were installed on the lane of the indoor track from 30 to 40 m to analyze running stride patterns during the maximum velocity phase. Ground contact time, flight time, step frequency, and stride length were measured during this section, which represented the maximal speed phase [19].

#### 2.3.3. Ratings of Perceived Exertion (RPE)

The 15-point (6–20) Borg scale [20] was used to record RPE. Participants were encouraged to use decimals, and the scale was “anchored” in a way that a previous memory of maximum exhaustion should equate to a score of 20. They were directly requested to report “how hard, heavy, and strenuous this repetition was” [21]. In this way, they were instructed to report the mental sense of effort generated by the task after each repetition.

#### 2.3.4. Core Affect

Dynamic changes in core affective state in participants were analyzed through the use of three different psychometric variables [14]. In this way, they were requested to indicate dynamic changes in valence from −5 (“very bad”) to 0 (“neutral”) to +5 (“very good”) after each 100 m repetition using the 11-point Feeling Scale (FS [22]). Participants also had to indicate felt arousal just before the first 100 m repetition and after each 100 m repetition through the 6-point Felt Arousal Scale (FAS [23]) from 1 (“low activation”) to 6 (“high activation”). Using decimals was recommended in order to rate felt arousal.

### 2.4. Statistical Analyses

Statistical analyses were performed using the Statistical Package for the Social Sciences 24.0 (IBM, Armonk, NY, USA). Data were checked for normality of distribution, equality of variances, and assumption of sphericity as appropriate. Greenhouse–Geisser corrections were used if the sphericity assumption was violated. Two-way (group × repetitions) repeated measures analysis of variance (ANOVA) was conducted on performance, biomechanical and perceptual variables with repeated contrast tests and Bonferroni’s post hoc tests conducted to identify changes between successive repetitions and between groups for each repetition, respectively. Means and CV% of performance, biomechanical and perceptual variables, recent performance in 1500 m, and recent performance achieved at respective events of each group were compared between groups using independent *t*-tests, Cohen’s *d* [24] effect sizes (ES), and 95% confidence intervals (95% CI). The same comparisons were conducted with performance, biomechanical and perceptual variables for each repetition between groups and between successive segments for each group where appropriate. Statistical significance was accepted at *p* < 0.05. Cohen’s *d* was considered to be either trivial (*d* < 0.20), small (0.21–0.60), moderate (0.61–1.20), large (1.21–2.00), or very large (2.01–4.00) [25]. Effect sizes of the ANOVA were calculated using eta partial squared (*η*_p_^2^). In both figures, differences between successive repetitions and between groups at each repetition have been indicated only when the effect size was moderate or larger (*d* ≥ 0.61) and the 95% CI did not cross zero.

## 3. Results

In Table 1, means and SD of performance, pacing, biomechanical and perceptual variables collected from both speed- and endurance-adapted milers and ES and 95% CI from comparison of these variables between groups are displayed. Both groups displayed similar recent performances in the 1500 m event (Table 1) and the only significant difference between groups was found between accumulated times achieved in the first five and last five 100 m repetitions. Therefore, although both groups displayed a positive pacing profile, it was more pronounced in the speed- than endurance-adapted milers (Table 1). However, despite displaying no significant differences, the rest of the variables showed either small or moderate ES. Speed-adapted milers performed faster repetitions and displayed a faster maximal speed, a lower flight time, contact time, and valence, and a higher step frequency, stride length RPE, and felt arousal than endurance-adapted milers (Table 1).

In Table 2, means and SD of CV% of performance, biomechanical and perceptual variables are displayed and comparisons between groups of these variables are shown. No significant differences were found between groups in either 1500 m performance or longer and shorter distances. A higher CV% in the speed- than in endurance-adapted milers with moderate ES was shown in the average of repetition times and with large ES in contact time and valence (Table 2).

**Figure 1:**
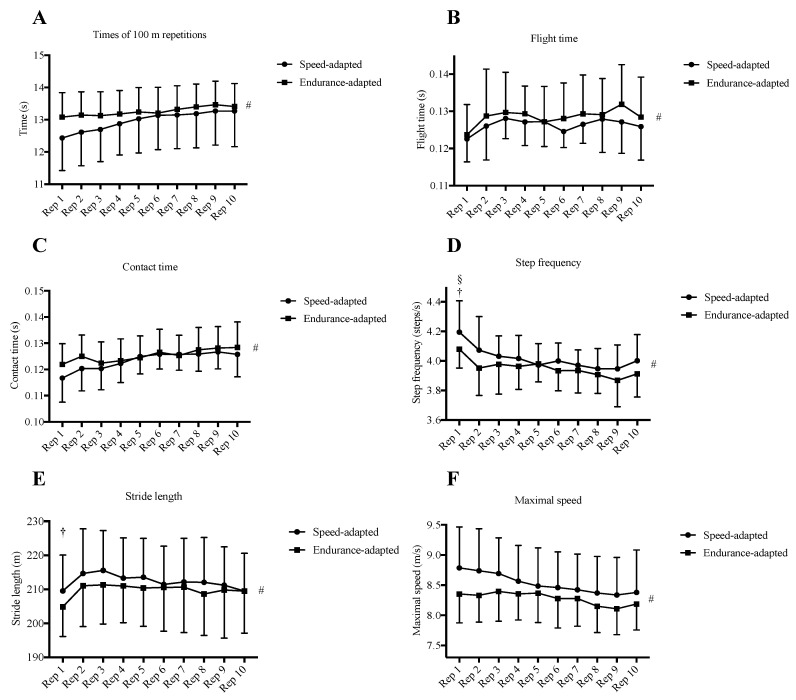
Mean (± SD) repetitions time (**A**), flight time (**B**), contact time (**C**), step frequency (**D**), stride length (**E**), and maximal speed of each repetition (**F**). † and § indicate the differences between successive repetitions (*p* < 0.05, *d* ≥ 0.61) in speed- and endurance-adapted milers, respectively. # indicates main time effect.

**Figure 2:**
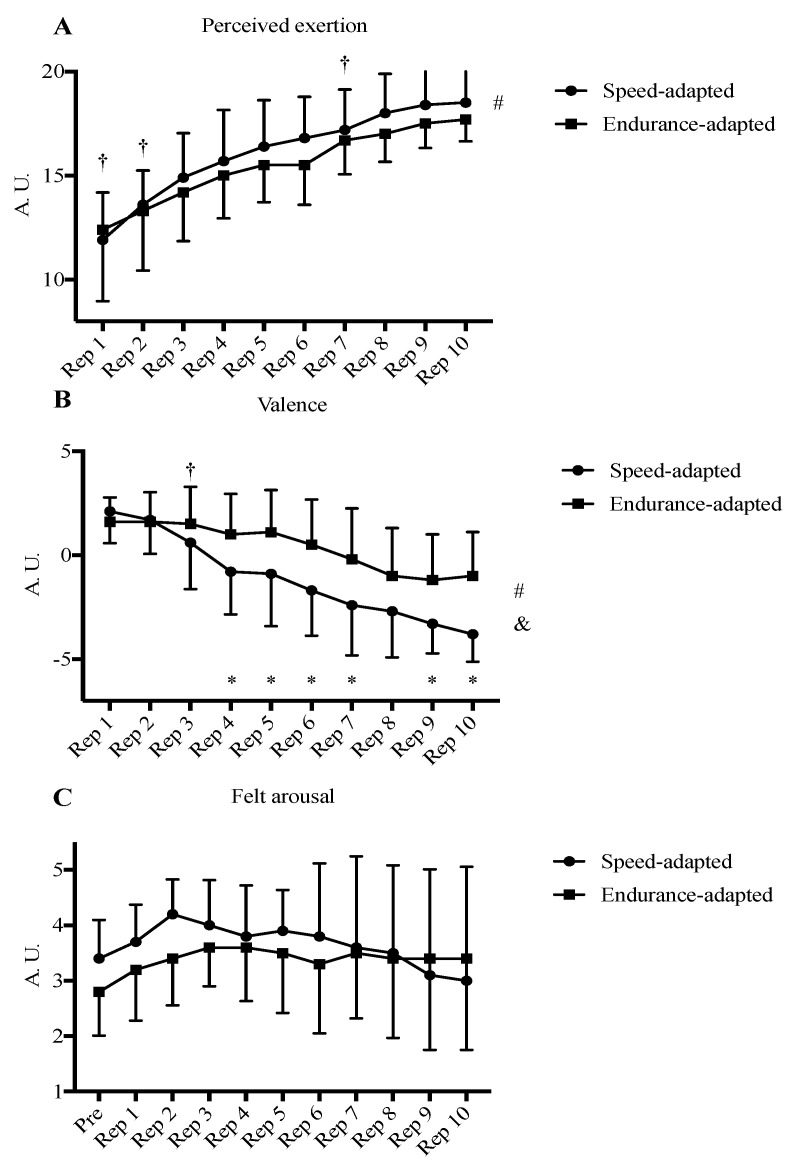
Mean (± SD) ratings of perceived exertion (**A**), valence (**B**) and felt arousal (**C**). † indicates the differences between successive repetitions (*p* < 0.05, *d* ≥ 0.61) in speed-adapted milers. * indicates the differences between groups after each repetition. # and & indicates main time and group effect, respectively.

Figure 1 shows the means and SD of performance and biomechanical variables measured following each repetition and Figure 2 shows the perceptual variables collected following each repetition. Significant time effects of the ANOVAs were found on the biomechanical and perceptual variables. Times of 100 m repetitions (*F*_2.49, 44.76_ = 25.53, *p* < 0.001, *η*_p_^2^ = 0.586), flight time (*F*_4.73, 85.2_ = 3.22, *p* = 0.012, *η*_p_^2^ = 0.152), contact time (*F*_5.4, 97.15_ = 21.2, *p* < 0.001, *η*_p_^2^ = 0.541), stride length (*F*_9, 172_ = 6.71, *p* < 0.001, *η*_p_^2^ = 0.271), and RPE (*F*_2.21, 39.72_ = 21.297.19, *p* < 0.001, *η*_p_^2^ = 0.844) increased across the session despite showing no significant time × group interaction effect or group effect (Figure 1A–C,E and Figure 2A, respectively), whereas step frequency (*F*_4.23, 76.06_ = 11.02, *p* < 0.001, *η*_p_^2^ = 0.38) and maximal speed (*F*_3.99, 71.77_ = 17.98, *p* < 0.001, *η*_p_^2^ = 0.5) decreased across the session despite showing no significant time × group interaction effect or group effect (Figure 1D,F, respectively).

Furthermore, the group × time interaction effect for valence was significant (*F*_3.08, 55.38_ = 6.07, *p* = 0.001, *η*_p_^2^ = 0.25). Ratings of affective valence decreased during the session and were higher in the endurance- than in the speed-adapted milers after the 4th (*p* = 0.033, *d* = 1.04, 95% CI = −3.82–−1.85), 5th (*p* = 0.04, *d* = 0.99, 95% CI = −4.29–−1.11), 6th (*p* = 0.022, *d* = 1.12, 95% CI = −4.41–−0.39), 7th (*p* = 0.037, *d* = 1.01, 95% CI = −4.64–−0.16), 9th (*p* = 0.01, *d* = 1.28, 95% CI = −3.99–−0.61), and 10th repetition (*p* = 0.001, *d* = 1.78, 95% CI = −4.56–−1.42) (Figure 2B).

## 4. Discussion

The main findings of this study were that elite speed-adapted milers displayed a more positive pacing profile, a lower affective valence and a greater change of ground contact time and average repetition time across a SIT session than endurance-adapted milers.

Whilst differences between groups were not observed in average repetition time, maximal speed, or biomechanical and perceptual variables, it is noteworthy that differences were found in the manner in which some of these variables changed across the session. Speed-adapted milers displayed both significantly higher variability of repetition time and difference among the accumulated times between the last and the first five repetitions than endurance-adapted milers (Table 2), displaying a more positive pacing pattern. Considering that this training session was designed whereby middle-distance runners should achieve their fastest speed in the first repetition with a subsequent decrease throughout, this finding was expected because speed-adapted milers displayed a faster recent performance in the 800 m event. It has been demonstrated that 800 m races are typically characterized by a positive pacing profile at both major championships [8,26,27] and during world record performances [9,28], whereas in 1500 m and longer races such as 5000 m are typically characterized by either a U-shaped parabolic pacing pattern during record performances [9] or an even pace with a fast end spurt during championships [8]. Furthermore, although no significant differences were found between repetition times or maximal speed between groups, speed-adapted milers displayed both faster times (Figure 1A) and maximal speed (Figure 1F) in the first three repetitions than endurance-adapted milers, which indicates their higher ability to generate a higher power output than endurance-adapted milers [10]. Conversely, endurance-adapted runners were able to maintain a more even pace across the session, which may also be explained by their higher ability to use aerobic metabolism [2]. In agreement with this finding, speed-adapted milers showed a higher variability in ground contact time during the training session than endurance-adapted milers (Table 2). Although this biomechanical variable does not differ between runners by level of performance [2], it does when the same athletes run at different velocities [29] and, therefore, it also may explain the higher variability of repetition time showed among groups during the session. In addition, and in agreement with other research [13], flight time (Figure 1B), contact time (Figure 1C) and stride length (Figure 1E) increased across repetitions whereas step frequency decreased during the session (Figure 1D). Similar biomechanical changes have been previously associated to a decrease of root mean square surface electromyography (EMG) activities of rectus and biceps femoris [30]. In addition, the loss of speed observed in this study was similarly reported previously in elite sprinters who conducted a sprint training session and was correlated with an increase in blood lactate and ammonia concentration, along with jump height loss during a countermovement jump [31]. Therefore, the higher ability of endurance-adapted milers to maintain a given speed might be related to their supposedly higher values of VO_2max_ and running economy, which may allow them to produce higher rates of reoxygenation and blood lactate clearance than speed-adapted milers [32]. It is noticeable that significant biomechanical changes were only found between the first and second repetition in step frequency in both groups (Figure 1D) and stride length in speed-adapted milers (Figure 1E). In this sense, these changes might represent the appearance of fatigue after the first repetition [33,34], and this early fatigue expression potentially resulting from a theoretically expected decrease in neuromuscular activation and an increase in blood lactate and ammonia concentration [30,31], which was also verified by rapid changes in perceptual ratings such as RPE and affective valence. Valence decreased across repetitions and was lower in speed-adapted milers than in endurance-adapted milers (Figure 2B) whereas RPE increased during the session and did not differ between groups (Figure 2A). Whilst RPE represents an indication of somatic stress and somatic strain [20], affective valence comprises the different feeling states that are experienced in a specific situation and this combination closely approximates to the individual’s evaluation and interpretation of these situations [15,35]. In this sense, it has been observed that responses in valence are related to awareness of performance [15]. The decrease in valence across the session may also be explained by an increase in peripheral physiological fatigue [15], however, a limitation of our study is that we have no peripheral physiological data from our participants. Whatever the mechanisms, this variability did not influence RPE (Figure 1A), probably because it relates to how the athlete feels rather than what the athlete feels, which in turn is rated by valence. Therefore, a dissociation of the response of these perceptual variables was observed. Furthermore, in the speed-adapted milers, the biggest changes in RPE occurred during the first three repetitions whereas in valence this occurred between the third and the fourth repetition (Figure 2A,B). Although the underpinning mechanisms involved remains unclear, it may be explained by the decrease of 100 m time between the first and fourth repetition in this group (Figure 1A) that may have been experienced by these runners, whereas RPE increased earlier due to the appearance of fatigue after covering the first 100 m repetition. In addition, no significant differences across repetitions or between groups were observed in felt arousal (Figure 2C), a similar observation to that made in a previous study also conducted with elite middle-distance runners comparing the perceptual responses when completing an interval training session individually and within a group of runners [36]. However, in speed-adapted milers felt arousal values were non-significantly higher than in endurance-adapted milers during the first three repetitions (Figure 2C). In this sense, Kilpatrick et al. [37] found that felt arousal was related to the intensity of the exercise and it would explain these differences in our study given that speed-adapted milers displayed a more positive pacing profile than endurance-adapted milers. A limitation of the present study is that due to the low number of female participants (i.e., four), we were not able to analyze sex differences in the variables studied. Unfortunately, only four female athletes belonged to the training group that was studied and we could not recruit other elite female middle-distance runners. Further studies could analyze these between-sex differences in biomechanical, perceptual, and performance responses during SIT in elite distance runners.

## 5. Conclusions and Practical Applications

The results of this study found that during a SIT, speed-adapted milers displayed a more positive pacing strategy than endurance-adapted milers. The faster decrease in speed demonstrated by speed-adapted milers resulted in a higher range of contact times and lower levels of affective valence throughout the session than those found in endurance-adapted milers. However, no differences in performance or in other biomechanical and perceptual measures were found between groups. Categorizing the different types of milers according to the distance that they either are training for or display higher predisposition to excel at has also not previously conducted and it represents a more acute approach in order to understand the underpinning mechanisms, which may explain the differing responses of athletes to a specific training stimulus. Coaches and athletes should be aware that in order to achieve the optimal performance during a SIT, middle-distance runners may need to fully display their own abilities that are related to the type of event they are adapted to. In this manner, speed-adapted milers may need to display a more positive pacing profile than endurance-adapted milers and, therefore, would experience lower levels of affective valence during a SIT.

## Figures and Tables

**Table 1:** Means and standard deviations (SD) of performance, biomechanical and perceptual variables collected to speed- and endurance-adapted milers. Effect sizes (ES), *p*-value and confidence intervals (95% CI) calculated from the comparison of these variables between groups.

Performance, Biomechanical and Perceptual Variables	Mean ± SD		ES	*p*	95% CI
	Speed-Adapted	Endurance-Adapted			
Performance (IAAF)	977.8 ± 132.65	980.9 ± 110.68	0.03	0.86	−118.14–111.94
Other perform (IAAF)	944.4 ± 164.98	1003.5 ± 127.6	0.4	0.38	−197.67–79.47
Times of 100 m rep (s)	12.97 ± 1.03	13.26 ± 0.72	0.33	0.47	−1.12–0.54
Maximal speed (m/s)	8.53 ± 0.62	8.28 ± 0.45	0.45	0.32	−0.27–0.76
Flight time (s)	0.126 ± 0.006	0.129 ± 0.01	0.31	0.49	−0.01–0.005
Contact time (s)	0.12 ± 0.007	0.13 ± 0.008	0.21	0.64	−0.09–0.005
Frequency (steps/s)	4.02 ± 0.13	3.95 ± 0.14	0.45	0.32	−0.07–0.19
Stride length (m)	212.28 ± 11.54	209.75 ± 11.79	0.22	0.63	−8.43–13.48
RPE	16.14 ± 1.86	15.48 ± 1.85	0.36	0.44	−1.08–2.4
Valence	−1.12 ± 1.7	0.29 ± 1.96	0.77	0.09	−3.13–0.31
Felt arousal	3.64 ± 0.87	3.37 ± 0.98	0.29	0.53	−0.61–1.13
Halves difference (s)	2.36 ± 0.93	1.03 ± 1.48	1.07	0.03	0.17–2.49

Performance (IAAF): International Association of Athletics Federations (IAAF) performance scores at 1500 m event; other perform: IAAF performance scores of each group at either shorter or longer events than 1500 m event, respectively. rep: repetition; frequency: step frequency; RPE: rate of perceived exertion; halves difference: difference between accumulated times registered from the first 5 and last 5 100 m repetitions; SD: standard deviations; ES: Cohen’s *d* effect size; *p*: *p*-value; CI: confidence intervals.

**Table 2:** Means and standard deviations (SD) of the coefficient of variation (CV%) of performance, biomechanical and perceptual variables collected to speed- and endurance-adapted milers. Effect sizes (ES), *p*-values and confidence intervals (95% CI) calculated from the comparison of these variables between groups.

Performance, Biomechanical and Perceptual Variables	Mean ± SD		ES	*p*	95% CI
	Speed-Adapted	Endurance-Adapted			
Times of 100 m rep (%)	2.47 ± 0.74	1.64 ± 0.7	1.15	0.02	0.15–1.5
Maximal speed (%)	2.66 ± 0.44	2.45 ± 0.63	0.39	0.4	−0.3–0.72
Flight time (%)	0.13 ± 0.006	0.13 ± 0.009	0.4	0.38	−0.79–1.97
Contact time (%)	3.79 ± 0.82	2.66 ± 0.93	1.29	0.01	0.31–1.95
Step frequency (%)	3.79 ± 0.97	2.83 ± 1.35	0.82	0.08	−0.14–2.06
Stride length (%)	2.29 ± 0.55	2 ± 0.62	0.5	0.28	−0.26–0.85
RPE (%)	13.32 ± 3.56	12.1 ± 6.32	0.24	0.6	−3.61–6.03
Valence (%)	67.03 ± 35.16	30 ± 20.67	1.28	0.01	9.93–64.13
Felt arousal (%)	28.35 ± 14.04	20.26 ± 14.43	0.57	0.22	−5.29–21.46

Rep: repetitions; RPE: rate of perceived exertion; SD: standard deviations; ES: Cohen’s *d* effect size; *p*: *p*-value; CI: confidence intervals.
